# Preterm birth is associated with an increased fundamental frequency of spontaneous crying in human infants at term-equivalent age

**DOI:** 10.1098/rsbl.2014.0350

**Published:** 2014-08

**Authors:** Yuta Shinya, Masahiko Kawai, Fusako Niwa, Masako Myowa-Yamakoshi

**Affiliations:** 1 Graduate School of Education, Kyoto University, Kyoto, Japan; 2 Department of Pediatrics, Graduate School of Medicine, Kyoto University, Kyoto, Japan; 3 Japan Society for the Promotion of Science, Tokyo, Japan

**Keywords:** preterm infants, spontaneous cry, fundamental frequency, low-birth-weight infants, small-for-gestational-age

## Abstract

Human infant crying has been researched as a non-invasive tool for assessing neurophysiological states at an early developmental stage. Little is known about the acoustic features of spontaneous cries in preterm infants, although their pain-induced cries are at a higher fundamental frequency (*F*_0_) before term-equivalent age. In this study, we investigated the effects of gestational age, body size at recording and intrauterine growth retardation (IUGR) on the *F*_0_ of spontaneous cries in healthy preterm and full-term infants at term-equivalent age. We found that shorter gestational age was significantly associated with higher *F*_0_, although neither smaller body size at recording nor IUGR was related to increased *F*_0_ in preterm infants. These findings suggest that the increased *F*_0_ of spontaneous cries is not caused by their smaller body size, but instead might be caused by more complicated neurophysiological states owing to their different intrauterine and extrauterine experiences.

## 1. Introduction

For several decades, acoustic features of infant crying have been studied as a possible non-invasive tool for assessing neurophysiological states [1–5]. Previous studies have indicated that an abnormally high frequency (*F*_0_) (e.g. mean *F*_0_ > 600 Hz) of infant cries is associated with medical conditions, including chromosomal, endocrine, metabolic and neurological disturbances at an early developmental stage [1]. Preterm birth is also a factor in higher *F*_0_ cries during early infancy [1–3]. Pain-induced cries in preterm infants have been reported to be higher in *F*_0_ before term-equivalent age compared with those of full-term newborns [2,3], although such differences disappeared around term-equivalent age [2,4]. A higher vocal *F*_0_ is generally related to smaller body size, especially shorter vocal folds [6]; therefore, it is possible that the higher *F*_0_ of preterm infants simply reflects premature body development.

Vocal *F_0_* also depends on a complex interaction between laryngeal and respiratory controls [1,6]. Specifically, vagal inputs from the right nucleus ambiguus of the medulla are assumed to have inhibitory effects on laryngeal muscle contraction and tightening of the vocal folds [5]. Therefore, diminished vagal activity might cause laryngeal muscle contraction and tightening of the vocal folds, resulting in a higher *F*_0_ [5]. As preterm infants exhibit reduced vagal activity even at term-equivalent age [7], the *F*_0_ of their cries might be affected by the altered vagal activity as well as smaller body size.

However, the effects of body size and other factors related to neurophysiological states (e.g. intrauterine growth retardation (IUGR)) on the *F*_0_ of cries in preterm infants have not been investigated [2–4]. In addition, although spontaneous cries (those unaffected by external acute stress) have a higher internal consistency than pain-induced cries in full-term neonates [8], to our knowledge, no studies have assessed the *F*_0_ of spontaneous cries in preterm infants. In this study, we performed acoustic analysis of the *F*_0_ of spontaneous cries before feeding in both healthy preterm infants at term-equivalent ages and full-term newborns. We investigated the effects of gestational age, body size at recording and IUGR on *F*_0_ to assess the relationship between preterm birth and the *F*_0_ of spontaneous cries at term-equivalent ages.

## 2. Material and methods

### (a) Participants

Forty-four healthy preterm infants (gestational age less than 37 weeks, weight at birth less than 2500 g) and 20 full-term (FT) newborn infants (gestational age more than or equal to 37 weeks, weight at birth more than or equal to 2500 g) were enrolled in this study. Preterm infants were assigned to two subgroups according to gestational age: very preterm (VP) infants (gestational age less than 32 weeks, *n* = 22) and moderate-to-late preterm (MLP) infants (gestational age between 32 and less than 37 weeks, *n* = 22). They were also grouped according to intrauterine growth: small for gestational age preterm (SGAP) infants (*n* = 19), and adequate for gestational age preterm (AGAP) infants (*n* = 25) (see the electronic supplementary material).

### (b) Cry recording and acoustic analysis

Cry recordings were performed at term-equivalent age (postmenstrual age between 37 and less than 42 weeks) at Kyoto University Hospital. The spontaneous cries of each infant in an open crib less than 30 min before feeding were recorded for 60 s using a wave recorder (EDIROL R-09; Roland Corp., Los Angeles, CA, USA) at a 44.1 kHz sampling rate and 16 bit-quantization. Cry utterances containing broad regions of environmental noise were excluded from acoustic analyses to avoid artefacts when determining *F*_0_. *F*_0_ measurements were performed using PRAAT v. 5.2.35 [9]. A total of 2321 cry utterances were extracted manually from the PRAAT spectrogram of all cry recordings for the acoustic analysis. The cry utterances were down-sampled to 22.05 kHz and low-pass filtered at 10 kHz to eliminate outliers and artefacts; then the minimum, mean and maximum *F*_0_ were determined using the PRAAT autocorrelation algorithm. The *F*_0_ values were averaged for each infant (see the electronic supplementary material for more information).

## 3. Results

One-way ANOVA of the gestational group revealed significant group differences in the minimum (*F*_2,61_ = 7.87, *p<*0.001, *η*^2^ = 0.10), mean (*F*_2,61_ = 9.17, *p* < 0.001, *η*^2^ = 0.23) and maximum *F*_0_ (*F*_2,61_ = 13.32, *p* < 0.00001, *η*^2^ = 0.30) (table 1). Post hoc testing (Bonferroni) revealed that the minimum (*p* < 0.0001) and mean *F*_0_ (*p* = 0.04) were significantly different between the VP and MLP groups. In addition, the minimum (*p* = 0.03), mean (*p* < 0.001) and maximum *F*_0_ (*p* < 0.0001) were significantly different between the VP and FT groups, whereas the maximum *F*_0_ (*p* = 0.01) was significantly different between the MLP and FT groups. We also examined the differences between the SGAP and AGAP groups using the two-tailed Student's *t*-test; however, there were no differences between the groups for any of the *F*_0_ variables (table 2).

**Table 1.** Difference in fundamental frequency (*F*_0_) of spontaneous crying in VP infants, MLP infants and FT infants.

	preterm						full-term						
	VP (*n* = 22)			MLP (*n* = 22)			FT (*n* = 20)						
	mean	s.d.	range	mean	s.d.	range	mean	s.d.	range	*F*-value	*p*-value	*η*^2^	post hoc (*p* < 0.05)
minimum *F*_0_ (Hz)	356	48	268–450	306	44	217–390	321	35	259–387	7.87	<0.001	0.10	VP > MLP, VP > FT
mean *F*_0_ (Hz)	458	47	381–548	425	40	348–491	403	38	318–463	9.17	<0.001	0.25	VP > MLP, VP > FT
maximum *F*_0_ (Hz)	539	59	460–642	511	44	435–609	460	44	361–524	13.32	<0.0001	0.32	VP > FT, MLP > FT

**Table 2.** Difference in fundamental frequency (*F*_0_) of spontaneous crying for SGAP infants and AGAP infants.

	preterm						*t*-value	*p*-value	*d*
	SGAP (*n* = 19)			AGAP (*n* = 25)					
	mean	s.d.	range	mean	s.d.	range			
minimum *F*_0_ (Hz)	320	55	217–411	340	49	246–450	1.27	0.21	0.39
mean *F*_0_ (Hz)	437	47	348–548	445	47	368–535	0.57	0.57	0.17
maximum *F*_0_ (Hz)	525	48	444–626	524	58	435–642	0.04	0.97	0.01

**Figure 1.**
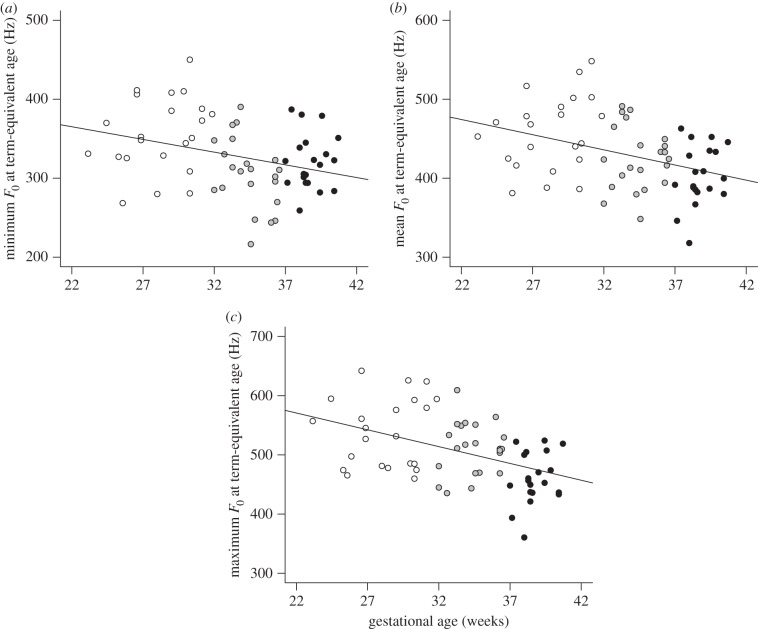
Scatter plots showing the relationships between gestational age and (*a*) minimum (*r*_S_ = −0.32, *p* < 0.01), (*b*) mean (*r*_S_ = −0.38, *p* < 0.01) and (*c*) maximum (*r*_S_ = −0.48, *p* < 0.0001) fundamental frequency (*F*_0_) of spontaneous cries at term-equivalent age for all participants (*n* = 64). The groups of infants were VP (white circles), MLP (grey circles) and FT (black circles).

Pearson's and Spearman's correlation analyses for all participants (*n* = 64) revealed that gestational age was significantly correlated with the minimum (*r*_S_ = −0.32, *p* < 0.01; figure 1*a*), mean (*r*_S_ = −0.38, *p* < 0.01; figure 1*b*) and maximum *F*_0_ (*r*_S_ = −0.48, *p* < 0.0001; figure 1*c*). However, infant weight at recording was marginally correlated with minimum *F*_0_ (*r*_S_ = 0.24, *p* = 0.06), but not mean (*r*_S_ = 0.11, *p* = 0.39) or maximum *F*_0_ (*r*_S_ = –0.01, *p* = 0.96). Other body size variables (height, head and chest circumference) at recording were not significantly correlated with any *F*_0_ variable (see the electronic supplementary material).

For multiple regression analyses, we used gestational age, postmenstrual age and weight at recording to avoid the collinearity of predictors due to strong correlations among the variables related to preterm birth or body size at recording. All *F*_0_ variables could be predicted by gestational age rather than postmenstrual age or weight at recording (table 3).

**Table 3.** The results of the multiple regression analysis predicting fundamental frequency (*F*_0_) of spontaneous crying. *β*, standardized regression coefficient; s.e., standard error.

	minimum *F*_0_			
predictor	*β*	s.e.	*t*-value	*p*-value
gestational age	−0.31	0.12	2.70	0.01
postmenstrual age	0.20	0.12	1.70	0.10
weight at cry recording	0.26	0.12	2.23	0.03
total model results	adjusted *R*^2^ = 0.20, *p* < 0.001			
	*F*_3,60_ = 6.19			

	mean *F*_0_			
predictor	*β*	s.e.	*t*-value	*p*-value
gestational age	−0.35	0.11	3.03	<0.01
postmenstrual age	0.29	0.12	2.46	0.02
weight at cry recording	0.11	0.12	0.97	0.33
total model results	adjusted *R*^2^ = 0.22, *p* < 0.001			
	*F*_3,60_ = 6.87			

	maximum *F*_0_			
predictor	*β*	s.e.	*t*-value	*p*-value
gestational age	−0.40	0.11	3.64	<0.001
postmenstrual age	0.32	0.11	2.87	0.01
weight at cry recording	0.01	0.11	0.07	0.95
total model results	adjusted *R*^2^ = 0.27, *p* < 0.0001			
	*F*_3,60_ = 8.95			

## 4. Discussion

This is the first study to determine the relationships between preterm birth and the *F*_0_ of spontaneous cries in human infants at term-equivalent age. We found that shorter gestational age was significantly associated with higher *F*_0_ of spontaneous cries; however, neither smaller body size at recording nor IUGR was related to increased *F*_0_ of spontaneous cries in preterm infants.

Preterm infants are usually smaller in body size than full-term infants at term-equivalent age. Therefore, the higher *F*_0_ of spontaneous cries may reflect the smaller body size at recording in preterm infants. However, we did not find any negative effects of body size at recording on any *F*_0_. As the relationship between body size and vocal folds is not strong, especially in the same age and sex class within species [10], it is possible that the body size measurements used in this study did not reflect individual differences in vocal fold size. By contrast, we found a weak positive effect of weight at recording on minimum *F*_0_. Vocal *F*_0_ is related to subglottic pressure from the lungs as well as the size of the vocal folds [6]. Therefore, higher subglottic pressure might increase the minimum *F*_0_ in infants with increased body weight at recording. As the MLP group had lower body weight at recording than the FT and VP groups (see the electronic supplementary material), the relatively low minimum *F*_0_ in the MLP group might have been due to their lower body weight at recording. However, our data at least suggest that the increased *F*_0_ of spontaneous cries in preterm infants is not due to their smaller body size at recording.

Nevertheless, it remains unclear why preterm birth is associated with an increased *F*_0_ of spontaneous cries at term-equivalent age. Our data did not provide a direct answer to this question. However, one possibility is that the increased *F*_0_ might reflect the reduced vagal activity in preterm infants. Vagal input has an inhibitory effect on laryngeal contraction and vocal fold tightening; therefore, diminished vagal activity is assumed to cause increased vocal fold tension and higher *F*_0_ [5]. As preterm infants at term-equivalent age exhibit lower resting vagal activity than full-term newborns [7], their reduced vagal activity might cause higher tension in the vocal folds, resulting in an increased *F*_0_ of spontaneous cries. To clarify this relationship, further studies involving direct investigation of vagal activity by measuring respiratory sinus arrhythmia are required.

An additional important point is that we included preterm infants who exhibited IUGR because no studies have assessed *F*_0_ of cries in SGA infants. As some preterm infants could exhibit IUGR, which can negatively affect neurobehavioural maturation [11], it is possible that this negative maturation might be related to the increased *F*_0_ of spontaneous cries in preterm infants. However, we were unable to identify the effects of IUGR on the *F*_0_ in preterm infants, suggesting that the increased *F*_0_ in preterm infants does not reflect a neurophysiological vulnerability specific to IUGR. Moreover, it is important to note that our reported *F*_0_ value in preterm infants did not necessarily deviate from the normal *F*_0_ range (i.e. 200–600 Hz) reported previously [1]. This might have been because we included low-risk preterm infants without severe complications. Nevertheless, we should investigate the effects of preterm birth in a study with stronger homogenization including full-term SGA infants.

It is also possible that the increased *F*_0_ of spontaneous cries in preterm infants is due to the longer postnatal period. This study did not include postnatal age in the major analyses because of a strong negative correlation between gestational and postnatal age. Nevertheless, each postnatal and postmenstrual age was positively correlated with every *F*_0_, even after controlling for gestational age (see the electronic supplementary material). In mammals, the production of a cry involves cortical regions including the anterior cingulate gyrus, as well as reflexive central pattern generators in the periaqueductal grey and nucleus retroambiguus [12,13]. Recent study of full-term infants shows that cry melodies (variation in *F*_0_ over time) become increasingly complex during early interaction with the environment in the first months of life [14], suggesting the early contribution of cortical control to the *F*_0_. However, it remains unclear in preterm infants at term-equivalent age; therefore, additional longitudinal studies will help to assess the effects of the postnatal and maturational period on the *F*_0_ in preterm infants.

In conclusion, this study revealed that preterm birth was associated with an increased *F*_0_ of spontaneous cries at term-equivalent age, regardless of the smaller body size at recording or IUGR. Hence, the increased *F*_0_ in preterm infants may have been caused not by smaller body size at recording, but rather by more complicated neurophysiological states due to different intrauterine and extrauterine experiences.

## Supplementary Material

Supplementary Materials
