# Supplementary material for: Preterm birth is associated with an increased fundamental frequency of spontaneous crying in human infants at term-equivalent age
Source: Biol Lett. 2014 Aug;10(8):20140350. doi: 10.1098/rsbl.2014.0350 (PMC4155907; doi:10.1098/rsbl.2014.0350)
Supplement: Supplementary Materials [file rsbl20140350supp1.doc]

**Electronic Supplementary Material (ESM)**

**Preterm birth is associated with an increased fundamental frequency of spontaneous crying in infants at term-equivalent age**

by

Yuta Shinya, Masahiko Kawai, Fusako Niwa, and

Masako Myowa-Yamakoshi*

*Corresponding author

E-mail: myowa.masako.4x@kyoto-u.ac.jp

This file includes:

- Supplementary text (Supplement S1-S5)

- References

- Demographic data for all participants (Table S1 and Table S2)

- Correlations between demographic and cry acoustic variables (Table S3)

**Supplement S1: *Participants***

We did not include infants with severe neurological complications such as brain lesions (including periventricular leukomalacia and Grade III or IV intraventricular haemorrhages), chromosomal abnormalities, bacterial meningitis, congenital hypothyroidism, herpes simplex viral encephalitis, or those who required intubation for respiratory disease during cry recordings. Intrauterine growth retardation of preterm infants was defined as a weight or height at birth below the tenth percentile for gestational age [1]. Table S1 shows the demographic characteristics of the very preterm (VP), moderate-to-late preterm (MLP), and full-term (FT) infant groups. Table S2 shows the demographic data for the small for gestational age preterm (SGAP) and the adequate for gestational age preterm (AGAP) infant groups. We did not acquire data regarding umbilical cord artery pH from seven preterm infants because an insufficient amount of material was collected.

**Supplement S2: *Cry recordings and acoustic analyses***

Preterm infants were recorded in a growing care unit at Kyoto University Hospital, where they stayed until leaving the hospital. Full-term infants were recorded in a quiet examination room. The noise level in the rooms was judged perceptually as low environmental noise without other infants crying, and was acceptable for audio recording and analysis. During recording, the distance between the microphone and the infant’s mouth was at 15 cm. A cry utterance was defined as a vocal output occurring on a single expiration [2] and lasting for at least 0.3 s to exclude non-cry sounds such as coughs. A total of 2,730 cries were extracted, and those that contained broad regions of environmental noise were excluded from the analysis to avoid artefacts when determining the F_0_. Ultimately, 2,321 cries (85.0% of all cry utterances) were used in the acoustic analyses (VP group, mean number of cries per infant = 35.5, range = 15–73; MLP group, mean number of cries per infant = 37.7, range = 18–71; FT group, mean number of cries per infant = 35.6, range = 14–67; SGAP group, mean number of cries per infant = 33.8, range = 18–65; AGAP group, mean number of cries per infant = 38.7, range = 15–73).

All acoustic measurements and manipulations were performed using PRAAT ver. 5.2.35 [3]. F_0_ measurements were made using a noise-resistant autocorrelation method at 150–900 Hz with a Hanning window length of 0.05 s. The terms and measures of the cry acoustic variables were defined as follows [4,5]: **Fundamental frequency (F_0_)** is the lowest-frequency component of a cry utterance, generally perceived as pitch, measured in Hz; **minimum F_0_** is the lowest fundamental frequency of a cry utterance; **mean F_0_** is the mean fundamental frequency of a cry utterance; **maximum F_0_** is the highest fundamental frequency of a cry utterance.

Minimum F_0_ of a vocalization is related to body size including vocal fold size among primate species [5]. Maximum F_0_ is related to body weight during crying in full-term neonates [2], as can be explained by diminished vagal activity due to acute pain stress in human infants [6]. The overall F_0_ of crying may be related to pathological conditions including preterm birth, chromosomal abnormalities, bacterial meningitis, congenital hypothyroidism, herpes simplex viral encephalitis, hyperbilirubinemia, and hypoglycaemia [7].

**Supplement S3: *Statistical analyses of group differences***

Group differences in the F_0_ of spontaneous cries among the VP (*n* = 22), MLP (*n* = 22), and FT (*n* = 20) groups were assessed using one-way ANOVA or Kruskal-Wallis tests followed by multiple comparisons using two-tailed Student’s *t*-tests or Mann–Whitney tests with a Bonferroni correction. Effect sizes, as represented by eta squared (*η^2^*), were also calculated for the main effects. Differences between the SGAP (*n* = 19) and AGAP (*n* = 25) groups were assessed using two-tailed Student’s *t*-tests or Mann–Whitney test. Effect sizes, as represented by Cohen’s *d*, were also calculated for the main effects.

**Supplement S4: *Internal consistency for cry acoustic variables***

To ensure internal consistency of the cry acoustic variables within each subject, we recorded 25% of all infants (16/64) twice during term-equivalent age (postmenstrual age between 37 and < 42 weeks). The times of any two cry recordings were separated by 1 or more weeks (mean days, 8.1; range, 7–14). High internal consistency was confirmed by significant positive correlations between two cry recordings (minimum F_0_, *r* = 0.83, *p* < 10^-4^; mean F_0_, *r_s_* = 0.74, *p* < 10^-3^; maximum F_0_, *r_s_* = 0.73, *p* < 0.01).

**Supplement S5: *Relationships between demographic and cry acoustic variables***

Pearson’s and Spearman’s correlations were calculated to assess the relationships between cry demographic and acoustic variables for all participants (*n* = 64), except for umbilical cord artery pH (*n* = 57) (Table S3). The demographic variables related to preterm birth (gestational age, Apgar score 1 and 5 min, weight at birth, postnatal age, and intubation period) and body size at recording (weight, height, and head and chest circumference) were significantly correlated with each other. In addition to the correlations reported in the main text, Apgar score 1 min, Apgar 5 min, postnatal age, postmenstrual age, and intubation period were significantly correlated with F_0_ values. However, after controlling for gestational age, only postnatal age (minimum F_0_, *r*_s_ = 0.26, *p* = 0.04; mean F_0_, *r* = 0.39, *p* < 0.01; maximum F_0_, *r* = 0.43, *p* < 10^-3^) and postmenstrual age (minimum F_0_: *r*_s_ = 0.33, *p* < 0.01; mean F_0_: *r* = 0.40, *p* < 0.01; maximum F_0_: *r* = 0.41, *p* < 10^-3^) remained significantly correlated.

***References***

1. Itabashi, K. et al. 2010 Introduction of new neonatal standard anthropometric measurements. *Nihonshounikagakkaizasshi* **114**, 1271–1293 (in Japanese).
2. Wermke, K. & Robb, M. P. 2010 Fundamental frequency of neonatal crying: does body size matter? *J. Voice* **24**, 388–394. (doi:10.1016/j.jvoice.2008.11.002)
3. Boersma, P. & Weenink, D. 2011 Praat: doing phonetics by computer (Version 5.2.46). [Computer program]. Retrieved from http://www.praat.org/.
4. Soltis, J. 2004 The signal functions of early infant crying. *Behav. Brain Sci.* **27**, 443–458– discussion 459–490. (doi:org/10.1017/S0140525X0400010X)
5. Ey, E., Pfefferle, D. & Fischer, J. 2007 Do age- and sex-related variations reliably reflect body size in non-human primate vocalizations? A review. *Primates* **48**, 253–267. (doi:10.1007/s10329-006-0033-y)
6. Porter, F. L., Porges, S. W. & Marshall, R. E. 1988 Newborn pain cries and vagal tone: parallel changes in response to circumcision. *Child Dev.* **59**, 495–505. (doi:org/10.2307/1130327)
7. Wasz-Höckert, O., Michelsson, K., & Lind, J. 1985 Twenty-five years of Scandinavian cry research. In *Infant crying: Theoretical and Research Perspectives* (eds B. M. Lester, & C. F. Z. Boukydis), pp. 83–104. New York, NY: Plenum Press.
